# Functional near-infrared spectroscopy as a diagnostic aid for stable schizophrenia

**DOI:** 10.3389/fpsyt.2025.1635854

**Published:** 2025-09-22

**Authors:** Suzhen Zhang, Ting Li, Liangliang Chen, Tongkuai Cong, Xinping Kuai, Yonggang Mu

**Affiliations:** ^1^ Division of Psychotic Disorders, Shanghai Mental Health Center, Shanghai Jiao Tong University School of Medicine, Shanghai, China; ^2^ Clinical Center for Psychotic Disorders, National Center for Mental Disorders, Shanghai, China; ^3^ Department of Psychiatry, Shanghai Changning Mental Health Center, Affiliated Mental Health Center of East China Normal University, Shanghai, China

**Keywords:** schizophrenia, cognition, diagnosis, fNIRS, VFT

## Abstract

**Objective:**

Cognitive impairment in schizophrenia (SCZ) is common, but the mechanism remains unclear. This study aimed to investigate whether brain activation during the functional near−infrared spectroscopy (fNIRS) verbal fluency test (VFT) task is associated with cognitive deficits and to evaluate the reliability of fNIRS as a clinical tool for diagnosing stable SCZ.

**Methods:**

A total of 45 stable SCZ patients and 30 healthy controls (HC) were included. Demographic information, Positive and Negative Symptom Scale (PANSS), and MATRICS Consensus Cognitive Battery (MCCB) were assessed. During VFT, hemodynamic responses in the frontotemporal cortex were monitored with fNIRS.

**Results:**

During VFT, individuals with SCZ demonstrated a reduced number of valid words, lower β value in channel 8, 25-26, 35–36 and 47-48, and decreased integral value (IV) in both the prefrontal lobe and bilateral temporal lobes. IV of the temporal lobes and the β value of channel 48 demonstrated sensitivity for diagnosis of SCZ, with an area under the receiver operating characteristic curve of 0.781 (95% CI: 0.667-0.896), and 0.762 (95% CI: 0.655-0.869), respectively. Moreover, IV of the temporal lobes correlated positively with multi-domain of cognition, including speed of processing, attention/vigilance, social cognition and MCCB total scores. The β value of channel 48 correlated positively with speed of processing.

**Conclusion:**

Our findings suggest that fNIRS may serve as a valuable clinical measure of cognition assessment, and IV of bilateral temporal lobes and β value of channel 48 can be used as candidate biomarkers to differentiate individuals with schizophrenia.

## Introduction

Cognitive impairments have been widely recognized as core pathological features of schizophrenia (SCZ) that contribute significantly to functional disability and demonstrating limited responsiveness to interventions (1). Neuropsychological investigations have consistently identified a broad range of cognitive deficits in SCZ encompassing impairments in attention, processing speed, working memory and executive functions (2–4). Longitudinal studies have established that cognitive decline precedes the onset of psychotic symptoms by nearly a decade (5, 6). Emerging evidence suggests accelerated cognitive aging in specific domains among individuals with psychotic spectrum disorders compared to the general populations (7, 8). Nevertheless, the underlying neurobiological mechanisms remain poorly characterized.

Functional near-infrared spectroscopy (fNIRS), a non-invasive optical neuroimaging modality, quantifies cortical hemoglobin dynamics through infrared light absorption variations (9). It is particularly valuable for assessment of regional neuronal activity in the prefrontal and superior temporal cortices- brain regions that have been consistently implicated in the neuropathology of SCZ (10, 11). During verbal fluency tasks (VFT), SCZ patients exhibit attenuated hemodynamic responses in frontotemporal regions (channels 10, 11, 17, 19, 21, 23–32, 34, 36, 38–42, and 44–52) compared to controls (12). Similarly, both individuals at clinical high risk for psychosis (CHR) and patients with first-episode SCZ demonstrated significantly reduced neural activation during the verbal fluency task (VFT), particularly in the bilateral inferior prefrontal gyrus, right middle temporal gyrus, and left dorsolateral prefrontal cortex(lDLPFC) (13). These findings suggest that VFT may serve as an indicator of disrupted neural processing underlying the cognitive deficits associated with SCZ.

Recent studies have demonstrated the diagnostic sensitivity of fNIRS’s across neuropsychiatric disorders. A cross-sectional study examining psychosis-spectrum trajectories (healthy controls, clinical high-risk, first-episode psychosis, chronic schizophrenia) revealed distinct spatiotemporal activation patterns across clinical stages (14). In several studies, researchers found two indexes obtained from fNIRS measured VFT activity, the integral value (IV) and centroid value (CV), which can be used for diagnosis of major psychiatric disorders. In a Tawain cohort examining 192 patients with psychotic disorder, researchers found the frontal CV at 54 seconds enabled the accurate distinction between patients with major depressive disorder at a rate of 80.0% (sensitivity:78.9%, specificity: 72.5%) and those with bipolar MDD (15). Furthermore, a combined fNIRS index of IV and CV achieved sensitivity in classifying individuals with SCZ from healthy controls (16).

In the present study, we used 52-channel fNIRS to measure the prefrontal and temporal cortical hemodynamics during VFT performance, and assessed clinical symptoms and MATRICS Consensus Cognitive Battery (MCCB) in stable SCZ patients and matched controls. The main aim of the study was to validate and extend previous reports by testing the relationships between brain activation during the fNIRS-VFT task and cognitive deficits and to evaluate the reliability of fNIRS as a clinical aid for diagnostic tools in stable schizophrenia. This study may advance the clinical application of fNIRS in assisting the diagnosis of SCZ and provide an objective measure to facilitate research on neural mechanisms research and treatment.

## Materials and methods

### Participants

45 SCZ patients were enrolled in this study from February, 2022 to September, 2024 in the Changning Mental Health Center of Shanghai City. The inclusion criteria for this study were: (1) age 18–65 years; (2) meeting the Diagnostic and Statistical Manual of Mental Disorders, 5th edition (DSM-5) for schizophrenia; (3) the Positive and Negative Syndrome Scale (PANSS) score ≤ 60 with a score of 3 or less on positive items (delusions, conceptual disorganization, hallucinatory behavior, grandiosity, or suspiciousness/persecution); (4) clinical stability, continuous antipsychotic treatment with stable dosage for at least 6 months before enrollment; (5) understand the experimental procedure; and (6) written informed consent. Exclusion criteria were (1) meeting DSM-5 criteria for other mental disorders; (2) exhibiting physical or mental unstable condition. During the same period, a sex- and age-matched control cohort (n=30) was recruited through community advertisements. Healthy controls underwent diagnostic interviews and evaluations to confirm the absence of any personal or family psychiatric history. The study was approved by the Ethics Review Committee of the Changning Mental Health Center of Shanghai City. In accordance with the Declaration of Helsinki, written informed consent was obtained from all participants before enrollment.

On the experimental day, SCZ patients were evaluated for psychopathology with the PANSS (17). Both patient and control participants completed the MATRICS Consensus Cognitive Battery to assess cognitive function across 7 domains: speed of processing, attention/vigilance, working memory, verbal learning, visual learning, reasoning and problem-solving, and social cognition (18). To ensure assessment validity, all assessments was evaluated by two well-trained psychiatrist who were blinded to the fNIRS study results.

### Verbal fluency task

Participants performed a standardized Chinese-language VFT consisting of a total of 160 seconds (19). Each trial consisted: (1) a 10-s pre-task baseline period, (2) a 30-s first repeated counting period, (3) a 60-s task period subdivided into three 20-s blocks and (4) a 70-s post-task baseline period (20). During the task period, participants were asked to form as many valid words as possible from three commonly used characters: “tian”(sky; Block 1), “da”(big; Block 2), and “bai”(while; Block 3), and 20 s for each character. The total number of word phrases generated by each participant will be recorded. During the test, participants were instructed to keep eyes open, sit upright and minimize their movements.

### fNIRS measurements

We employed a 52-channel fNIRS system (ETG-4100, Hitachi Medical Co., Japan) to record evoked cortical activity during the VFT task (21). The ETG-4100 comprised 17 emitter locations and 16 light detectors arranged in a 3×11 matrix with a 3.0 cm inter-optode distance. This configuration was positioned according to the international 10–20 EEG system to cover the prefrontal and bilateral temporal cortex. Specifically, detector No. 26 was aligned over the glabella, ensuring the bottom edge of the fNIRS probe holder remained parallel to the eyebrows and coincident with the Fp1-Fp2 line. Each adjacent emitter-detector pair formed one of the 52 measurement channels, with the system operating at a sampling rate of 10 Hz. Each emitter delivered two wavelengths of near-infrared light (695 nm and 830 nm), which penetrated the scalp and skull to interact with cortical tissue. The detectors captured the optical signals, and using the modified Beer-Lambert law, the system calculated changes in the relative concentrations of oxygenated hemoglobin (oxy-Hb) and deoxygenated hemoglobin (deoxy-Hb).

### VFT data processing

The ETG-4100 system automatically performs post-task integral analysis following the VFT. In this procedure, the pre-task baseline was set as the last 10 seconds of the preceding 30-second interval, while the post-task baseline was defined as the first 55 seconds of the following 70-second interval. There are two key indexes reflecting the temporal midpoint of the fNIRS signal: the IV and the CV. They were calculated with 5-second moving average method (22). The IV measures the cumulative magnitude of the hemodynamic response across the entire 60-s task activation phase, while the CV indicates the temporal midpoint of the fNIRS signal change, covering both the task and post-task intervals. In line with prior research, the converted hemoglobin concentration data was utilized instead of raw optical intensity data to maintain the relationship between IV and CV in post-processing stage (21). Subsequently, the converted hemoglobin concentration changes were analyzed utilizing the NIRS-KIT software package within MATLAB R2013b (23). VFT data were directly obtained from Hitachi ETG4100 systems.

The original fNIRS data were preprocessed by preprocessed using the NIRS-KIT package. To eliminate slow time drifts, a first-order detrending procedure was applied. Next, motion correction was conducted by the correlation-based signal improvement method. Subsequently, artifacts were removed by applying a band-pass filter to restrict the frequency range of the data to 0.01–0.08 Hz (24). Finally, a general linear regression model (GLM) analysis was conducted to model the oxyhemoglobin response during the stimulus condition, with the aim of estimating the β coefficient as an indicator of individual task-related neural activity (23).

### Statistical analysis

Statistical analyses were performed using SPSS 22.0 (SPSS Inc., Chicago, IL, USA) and R Studio 4.1(RStudio Team, 2022). Continuous variables, including age, education(years), IV, CV, and β values for each channel, underwent Shapiro-Wilk tests to confirm normality. Normally distributed data were expressed as mean ± standard deviation (mean ± SD), while non-normally distributions were reported with interquartile range (IQR) as [M (Q1, Q3)]. Chi-square test was used to analyze the inter-group differences of gender. Internal channel activation was determined through one-sample analyses: parametric t-tests (H0: β=0). Intergroup channel activation comparisons (SCZ vs HC) employed independent t-tests. Forward stepwise logistic regression was adopted to identify potential features associated with diagnosis of SCZ. Model performance was assessed using receiver operating characteristic (ROC) curve analysis, with area under the curve (AUC). Pearson correlation analysis was employed to explore the associations between fNIRS features and cognitive performance.

## Results

### Demographic and clinical characteristics

No significant group differences were observed in age, sex and education (p>0.05; **Table 1**). Compared with controls, the stable SCZ group exhibited significant lower scores in MCCB total and four subdomains: speed of processing, attention/vigilance, reasoning and problem-solving, and social cognition (p<0.05).

**Table 1:** Demographic and clinical characteristics.

Variable	SCZ N=45	HC N=30	t/χ^2^(p)
Age (years)	42.78 ± 6.88	40.57 ± 8.47	1.242 (0.218)
Sex (M/F)	21/24	15/15	0.800 (0.777)
Education (years)	14.53 ± 2.62	15.23 ± 2.92	-1.083 (0.282)
PANSS-total	47.12 ± 9.33	NA	NA
PANSS-P	10.71 ± 3.34	NA	NA
PANSS-N	14.33 ± 4.64	NA	NA
PANSS-G	22.24 ± 3.90	NA	NA
MCCB (T-score)	40.44 ± 11.48	49.62 ± 9.53	3.579 (0.001)
Speed of processing	39.36 ± 13.09	53.83 ± 9.32	5.592 (<0.001)
Attention/vigilance	41.44 ± 10.73	49.55 ± 10.76	3.169 (0.002)
Working memory	47.69 ± 10.39	46.41 ± 8.33	-0.555 (0.580)
Verbal learning	37.33 ± 10.41	39.38 ± 9.00	0.869 (0.388)
Visual learning	51.29 ± 12.70	52.21 ± 9.65	0.332 (0.741)
Reasoning and problem-solving	46.24 ± 8.40	55.45 ± 8.84	4.507 (<0.001)
Social cognition	45.04 ± 12.37	51.00 ± 10.72	2.195 (0.032)

SCZ, schizophrenia; HC, healthy control; PANSS, Positive and Negative Syndrome Scale, which includes positive symptoms, negative symptoms and general psychopathology subscales; MCCB, Measurement and Treatment Research to Improve Cognition in Schizophrenia (MATRICS) Consensus Cognitive Battery.

### β value comparison between stable SCZ and HC groups in VFT

Examining the activation of each channel during the VFT showed different neural activation between the stable SCZ and HC groups. The HC group exhibited significant hemodynamic activation (β>0, pFDR<0.05) in 27 channels: 7-9, 14, 16-21, 24-29, 31, 35-39, and 46-50. In contrast, the stable SCZ group demonstrated activation in only 8 channels (16, 27-29, 38-39, and 40-50). Between-group comparisons identified significant β-value reductions in SCZ patients across key prefrontal regions (channels 8, 25–26, 35–36, and 47–48; pFDR<0.05) (**Table 2**).

**Table 2:** Comparison of β value between stable SCZ and HC groups in VFT.

Channel	Region	SCZ	HC	t (p)	Cohen’s d
8	lDLPFC	0.003 ± 0.013	0.012 ± 0.014	2.810 (0.006)	0.665
25	rDLPFC	0.002 ± 0.015	0.014 ± 0.020	3.001 (0.004)	0.665
26	mPFC	0.004 ± 0.016	0.014 ± 0.016	2.785 (0.007)	0.707
35	rVLPFC	0.001 ± 0.013	0.016 ± 0.022	3.425 (0.001)	0.891
36	mPFC	0.004 ± 0.015	0.014 ± 0.017	2.812 (0.006)	0.663
38	mPFC	0.008 ± 0.017	0.023 ± 0.022	3.332 (0.001)	0.785
47	mPFC	0.003 ± 0.015	0.017 ± 0.020	3.317 (0.001)	0.782
48	mPFC	0.004 ± 0.013	0.020 ± 0.018	4.019 (<0.001)	1.008

SCZ, schizophrenia; HC, healthy control; lDLPFC, left dorsolateral prefrontal cortex; rDLPFC, right dorsolateral prefrontal cortex; mPFC, medial prefrontal cortex; rVLPFC, right ventrolateral prefrontal cortex.

### Comparison of VFT performance, IV and CV between groups

The HC group demonstrated superior VFT performance in terms of total number of valid words (**Table 3**). Regarding the IV, significant differences were observed in both the prefrontal lobe and bilateral temporal lobes (p<0.001) when comparing the HC and SCZ groups, with the SCZ group exhibiting lower scores than the HC group (**Table 3**). In terms of CV, no notable differences were found in either the frontal lobe or the bilateral temporal lobes between the two groups (all p>0.05).

**Table 3:** Differences in VFT performance, IV and CV between groups.

Indicators	SCZ	HC	t/Z(p)	Effect size
VFT performance	8.98 ± 3.22	11.97 ± 3.44	3.817 (<0.001)	0.761^a^
IV of the prefrontal lobe	25.40 (-3.00,66.90)	57.70 (35.30,77.00)	-2.742 (0.006)	0.316^b^
CV of the prefrontal lobe	59.59 ± 15.33	56.43 ± 8.80	-1.022 (0.310)	-0.241^a^
IV of the Bilateral temporal lobes	28.00 (-1.55, 65.20)	110.10 (49.98, 157.50)	-4.110 (<0.001)	0.475^b^
CV of the Bilateral temporal lobes	58.66 ± 15.21	59.67 ± 7.65	0.340 (0.735)	0.080^a^

SCZ, schizophrenia; HC, healthy control; IV, integral value; CV, centroid value; ^a^ Cohen’s d is calculated for the effect size; ^b^ r is calculated for the effect size.

### Predictive value of fNIRS index on classification of HC and SCZ

**Figure 1:**
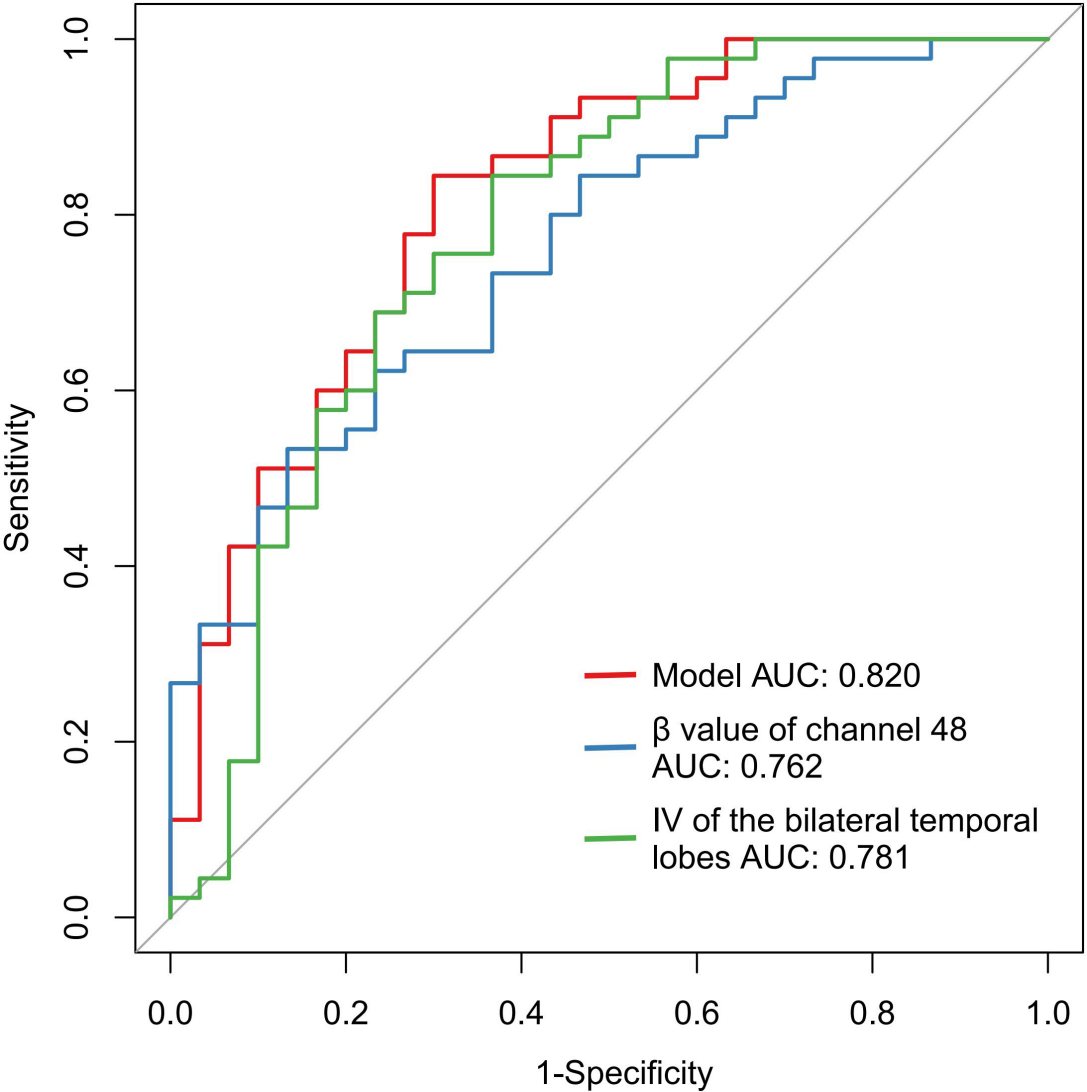
ROC for fNIRS features and combined index for the classification of SCZ.

**Figure 2:**
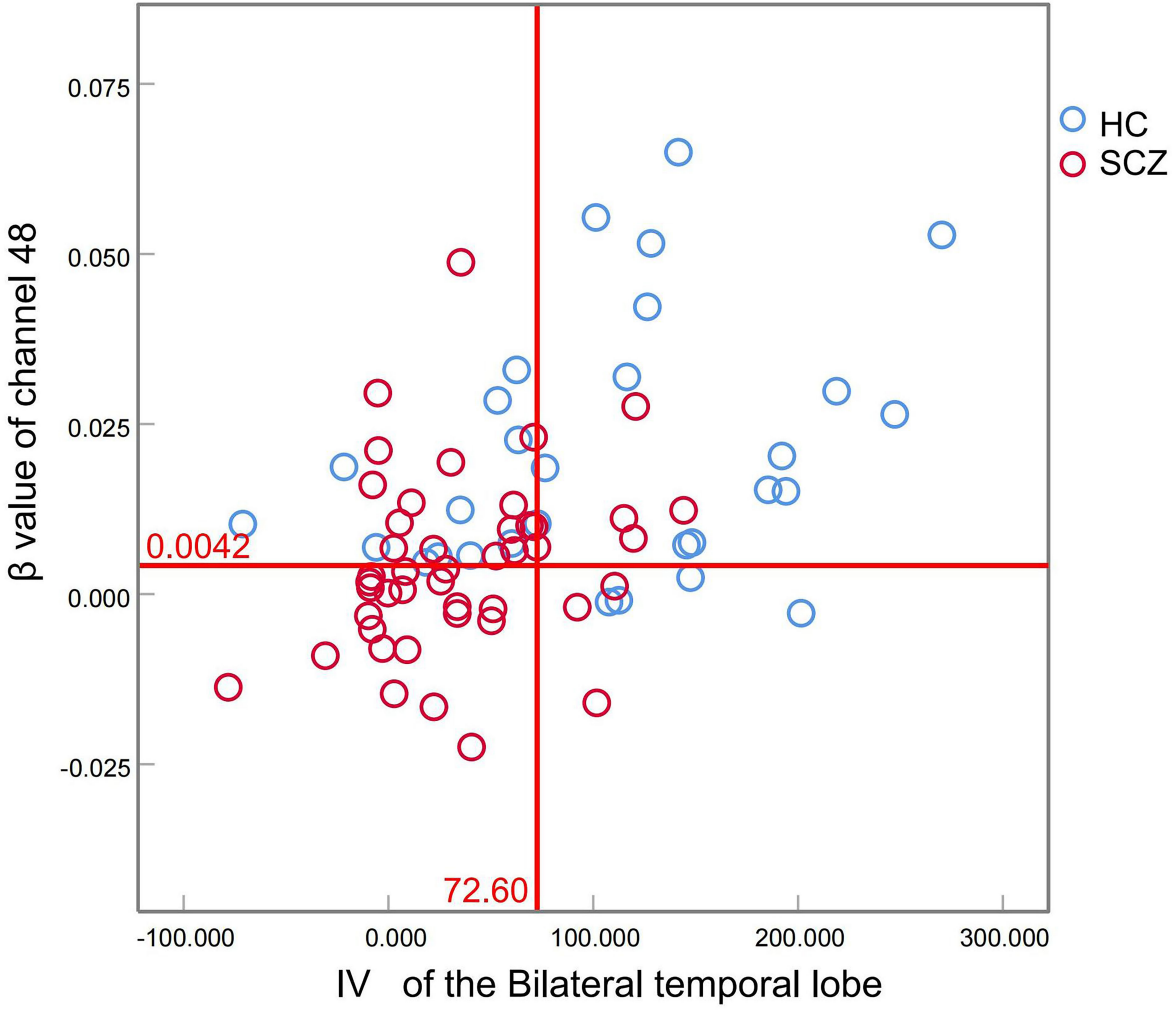
Scatter plots of IV of the bilateral temporal lobes and β value of channel 48 for each group.

Binary logistic regression analysis revealed that IV from bilateral temporal regions and β-values from channel 48 served as significant neurophysiological predictors for schizophrenia diagnosis (**Table 4**). Diagnostic performance evaluation demonstrated distinct discriminatory capacities: IV of the bilateral temporal lobes achieved an AUC of 0.781, and the β value of channel 48 yielded an AUC of 0.762 (**Table 5**, **Figure 1**). Combined implementation of these biomarkers provided an AUC of 0.820 (95%CI:0.720-0.920). Using the optimal feature thresholds, scatter plots for IV of the bilateral temporal lobes and β value of channel 48 for each group are shown in **Figure 2**.

**Table 4:** Logistic regression for predicting the patients with SCZ from HC.

Predictors in the model	Beta	S.E	P	OR	95%CI	
IV of the bilateral temporal lobes	-0.014	0.005	0.004	0.986	0.976	0.995
β value of channel 48^a^	-0.051	-0.022	0.020	0.951	0.911	0.992

SCZ, schizophrenia; HC, healthy control; IV, integral value; ^a^ Expand by 10^4 times.

**Table 5:** The diagnostic performance in distinguishing the patients with SCZ from HC by ROC analysis.

Indicators	AUC	p	95%CI	Cutoff	Sensitivity	Specificity
IV of the bilateral temporal lobes	0.781	<0.001	[0.667, 0.896]	72.60	0.633	0.844
β value of channel 48	0.762	<0.001	[0.655, 0.869]	0.0042	0.867	0.533
Model	0.820	<0.001	[0.720-0.920]	0.589	0.700	0.844

SCZ, schizophrenia; HC, healthy control; IV, integral value.

### Correlation between fNIRS features and cognition

**Figure 3:**
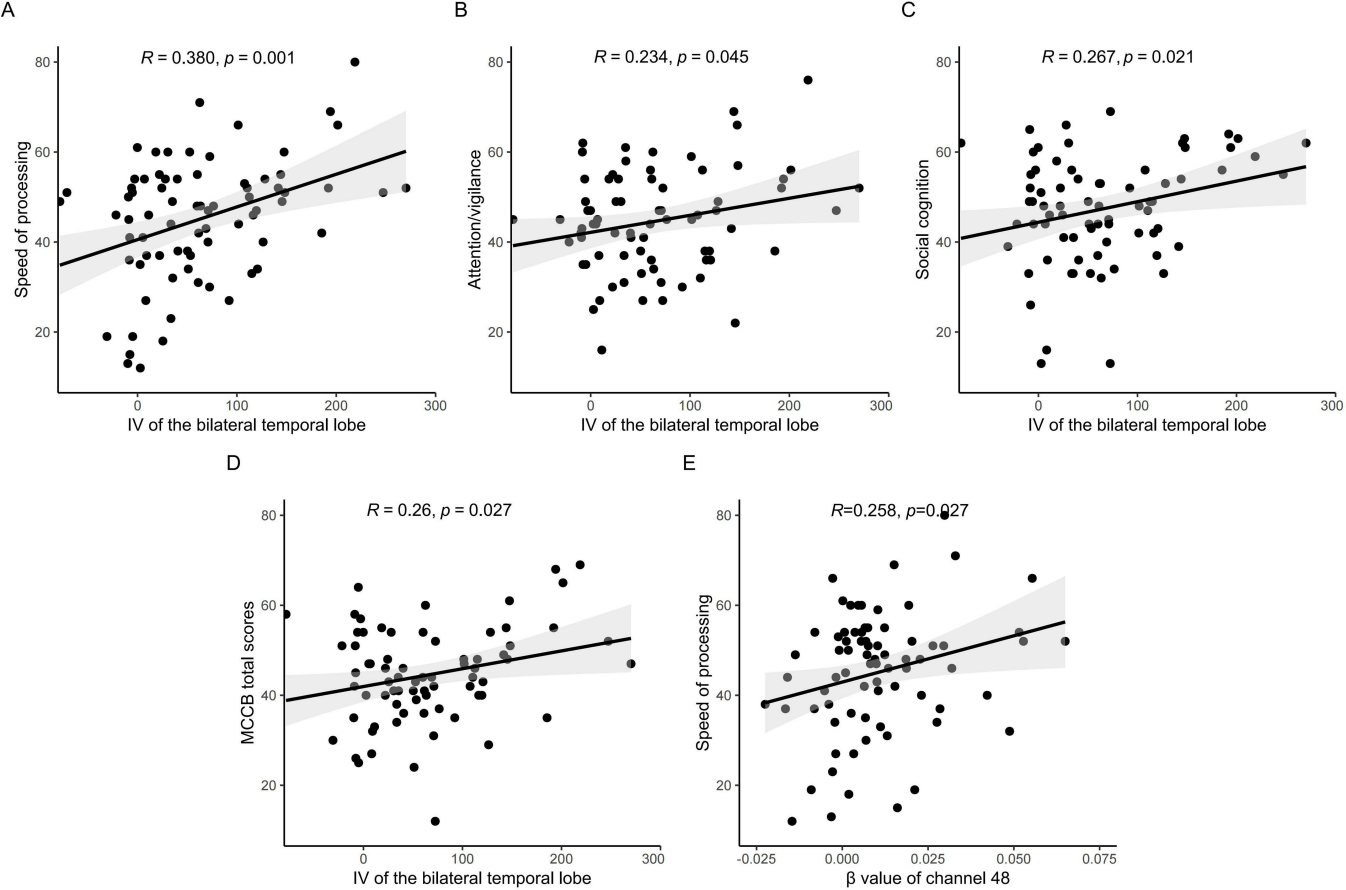
Correlation analysis between fNIRS features and cognition. IV of the bilateral temporal lobes correlated positively with processing speed **(A)**, attention/vigilance **(B)**, social cognition **(C)**, and MCCB total scores **(D)**, The β value of channel 48 correlated positively with speed of processing **(E)**.

Pearson correlation analyses revealed significant positive associations between IV of the bilateral temporal lobes and multiple cognitive domains: processing speed (r = 0.380, p = 0.001), attention/vigilance (r = 0.234, p = 0.045), social cognition (r = 0.267, p = 0.021), and MCCB total scores (r = 0.260, p = 0.027) (**Figure 3**). The β value of channel 48 correlated positively with speed of processing (r=0.258, p=0.027).

## Discussion

For many years, electroencephalography (EEG), fNIRS, and functional magnetic resonance imaging (fMRI) have been employed to investigate the functional activity of the cerebral cortex. EEG and fNIRS offer several advantages over fMRI in the study of dynamic brain activity: they are portable, cost-effective, and characterized by higher temporal resolution compared to fMRI (25). EEG exhibits high temporal resolution, and it is particularly susceptible to motion artifacts. In contrast, fNIRS provides distinct advantages including tolerance to motion artifacts, and user-friendly operational requirements (26). fNIRS, a state-of-the-art non-invasive brain functional imaging technology to detect the cortical oxygenation activity in real-time, is widely used in psychiatric populations (27, 28).

The VFT task, considered the gold-standard cognitive probe for fNIRS studies, effectively engages in the ability to retrieve and produce verbal material from the lexico-semantic memory, and to test executive control ability, such as processing speed, working memory, response inhibition and tasks requiring cognitive flexibility (29). The VFT performance activates and crucially relies on the superior medial frontal cortex, ventrolateral prefrontal cortex, and anterior temporal lobe (30). Poor performance on the VFT has been consistently linked to impairments in verbal and working memory, as well as executive functioning and problem-solving abilities in mental illness (31). Many studies have reported the brain dysfunction in patients with mental illness during fNIRS-VFT task. In patients with SCZ, reduced brain activation in the prefrontal cortex and superior temporal cortex was reported (32). Individuals with clinical high risk for psychosis demonstrate abnormal brain activation of rSTG (16). Crucially, differences in the performance of the fNIRS-VFT has been used to distinguish various psychiatric disorders. The present study investigated brain activation deficits during the Chinese version VFT using a 52-channel fNIRS device in stable SCZ and healthy controls, as well as evaluated the potential of these brain activation deficits as a clinical aid for diagnostic tools in SCZ.

Compared with HC, our study revealed that stable SCZ patients demonstrated poorer performance in MCCB total scores and cognitive subdomains (speed of processing, attention/vigilance, reasoning and problem-solving, and social cognition). These results may reflect impaired brain inefficiency in SCZ. Our findings align with previous studies demonstrating widespread cognitive dysfunction in SCZ patients across MCCB domains (3, 8). In this study, the SCZ group showed reduced activation values primarily in the mPFC and other regions including rVLPFC, rDLPFC, and lDLPFC relative to controls. Moreover, task-related β values were positive correlated with speed of processing. Our results are in line with previous studies that reduced resting cerebral blood flow and regional glucose metabolism in the PFC of SCZ patients (33). Findings from TMS/hd‐EEG measurements also indicate that intrinsic defects in both activity and connectivity of PFC in SCZ, and that these defects are specifically associated with cognitive impairment (34). Moreover, the PFC plays a critical role in regulating information processing speed and working memory (35). Hypofrontality in DLPFC has been linked to cognitive deficits in SCZ, and neuromodulation using rTMS over DLPFC has shown potential for improving cognitive function (36). Similar results were also found in EGG studies. Compared with controls, distinct functional connectivity patterns involving a wide range of brain regions- including the inferior, superior, and middle temporal gyri, the fusiform gyrus, the superior as well as the middle frontal gyri -were observed in EEG study of patients with chronic SCZ (37). In addition, previous studies have demonstrated that patients with schizophrenia exhibit reduced cortical inhibition in the motor cortex and DLPFC, with these deficits frequently correlating with symptom severity (38, 39).

Specially, a pronounced reduction in IVs in the frontal and bilateral temporal lobes observed during the VFT in the SCZ group, while no significant inter-group differences were noticed in CV values in our study. The IV of the bilateral temporal lobes exhibited positive correlations with processing speed, attention/vigilance, social cognition, and MCCB total scores. In a global ENIGMA study, convergent functional and structural epicenters across all stages of SCZ were predominantly localized within transmodal regions, including the parietal, temporal, and frontal lobes (10). Hypo-connectivity of the default-mode network (DMN) may reflect the impaired integration of inner activity, thereby offering a potential mechanistic explanation for the reduced IV in SCZ (40). Consistent with previous studies, the above results indicate that fNIRS may serve as a valuable clinical tool for assessing cognition in schizophrenia. The fNIRS technology has been widely utilized to detect biomarkers and evaluate cognitive impairments in various mental disorders. For instance, in major depressive disorder, both verbal learning and working memory positively correlated with functional connectivity of the bilateral frontotemporal cortex (41). During the Trial Making Test the Mazes Test, the mean changes in relative oxyhemoglobin concentration demonstrated negative correlations with PFC activation in the SCZ patients (31).

The IV measures the total hemodynamic response during the 60-s activation phase, and this biomarker has demonstrated diagnostic utility in mood disorders, particularly for differentiating depression subtypes (22). In a study involving ultra-high risk for psychosis individuals, first-episode psychosis, chronic schizophrenia, and healthy controls, IV was a significant biomarker for differentiating psychosis spectrum in various clinical stages with AUC range from 0.511 to 0.633 (p<0.05) (14). In a Chinese clinical population, temporal lobe IV was used to assist the diagnosis of major psychiatric disorders with an AUC of an 0.275, and the AUC improved to 0.923 with combined IV and CV (20). The former two studies indicated the combined use of IV and CV could improve the diagnostic sensitivity. In our results, IV of the bilateral temporal lobes alone distinguished patients from healthy controls with an AUC of 0.781(sensitivity 0.633, specificity 0.844). No significant reduction in CV was observed in stable SCZ patients in this study, suggesting preserved cortical homeostasis following task completion in our SCZ cohort. Notably, task-related β value of channel 48 is valuable in diagnosing SCZ from HC with an AUC of 0.762(sensitivity 0.867, specificity 0.533). Integration of the bilateral temporal IV and β value of channel 48 provided an AUC of 0.820. This may preliminarily suggest the high accuracy and reliability of using fNIRS as a clinical aid for diagnostic tools in SCZ.

Our findings not only highlight the distinct neurofunctional signatures in frontotemporal circuitry in SCZ but also validate fNIRS as a non-invasive and practical clinical tool for cognitive assessments. Nevertheless, all findings must be interpreted with caution as this study has several limitations. Deep learning techniques have demonstrated substantial value in fNIRS research aimed at diagnosis of SCZ, particularly in studies involving limited sample sizes (42, 43). In the current study, the SCZ group only included 45 individuals and further studies with deep learning techniques are required to confirm our results. Second, we only observed the cross-sectional outcome between the groups; the longitudinal changes in cognitive function and brain functional connectivity of SCZ are still not fully understood. Third, potential pharmacological confounders persist as patients maintained heterogeneous antipsychotic regimens, disease stages, highlighting the need for subgroup analyses.

## Conclusion

In conclusion, our findings suggest that fNIRS may serve as a valuable clinical measure of cognition assessment, and IV of bilateral temporal lobes and β value of channel 48 can be used as candidate biomarkers to differentiate individuals with schizophrenia.

## Data Availability

The raw data supporting the conclusions of this article will be made available by the authors, without undue reservation.
